# Apple Detection via Near-Field MIMO-SAR Imaging: A Multi-Scale and Context-Aware Approach

**DOI:** 10.3390/s25051536

**Published:** 2025-03-01

**Authors:** Yuanping Shi, Yanheng Ma, Liang Geng

**Affiliations:** 1 Department of UAV Engineering, Shijiazhuang Campus, Army Engineering University, Shijiazhuang 050003, China; 1102061@sjzc.edu.cn; 2 College of Mechanical and Electrical Engineering, Shijiazhuang University, Shijiazhuang 050035, China; liang_geng@bupt.edu.cn; 3 Shijiazhuang Key Laboratory of Agricultural Robotics Intelligent Perception, Shijiazhuang 050035, China

**Keywords:** fruit detection, synthetic aperture radar, millimeter-wave imaging, deep learning, precision agriculture

## Abstract

Accurate fruit detection is of great importance for yield assessment, timely harvesting, and orchard management strategy optimization in precision agriculture. Traditional optical imaging methods are limited by lighting and meteorological conditions, making it difficult to obtain stable, high-quality data. Therefore, this study utilizes near-field millimeter-wave MIMO-SAR (Multiple Input Multiple Output Synthetic Aperture Radar) technology, which is capable of all-day and all-weather imaging, to perform high-precision detection of apple targets in orchards. This paper first constructs a near-field millimeter-wave MIMO-SAR imaging system and performs multi-angle imaging on real fruit tree samples, obtaining about 150 sets of SAR-optical paired data, covering approximately 2000 accurately annotated apple targets. Addressing challenges such as weak scattering, low texture contrast, and complex backgrounds in SAR images, we propose an innovative detection framework integrating Dynamic Spatial Pyramid Pooling (DSPP), Recursive Feature Fusion Network (RFN), and Context-Aware Feature Enhancement (CAFE) modules. DSPP employs a learnable adaptive mechanism to dynamically adjust multi-scale feature representations, enhancing sensitivity to apple targets of varying sizes and distributions; RFN uses a multi-round iterative feature fusion strategy to gradually refine semantic consistency and stability, improving the robustness of feature representation under weak texture and high noise scenarios; and the CAFE module, based on attention mechanisms, explicitly models global and local associations, fully utilizing the scene context in texture-poor SAR conditions to enhance the discriminability of apple targets. Experimental results show that the proposed method achieves significant improvements in average precision (AP), recall rate, and F1 score on the constructed near-field millimeter-wave SAR apple dataset compared to various classic and mainstream detectors. Ablation studies confirm the synergistic effect of DSPP, RFN, and CAFE. Qualitative analysis demonstrates that the detection framework proposed in this paper can still stably locate apple targets even under conditions of leaf occlusion, complex backgrounds, and weak scattering. This research provides a beneficial reference and technical basis for using SAR data in fruit detection and yield estimation in precision agriculture.

## 1. Introduction

**Figure 1:**
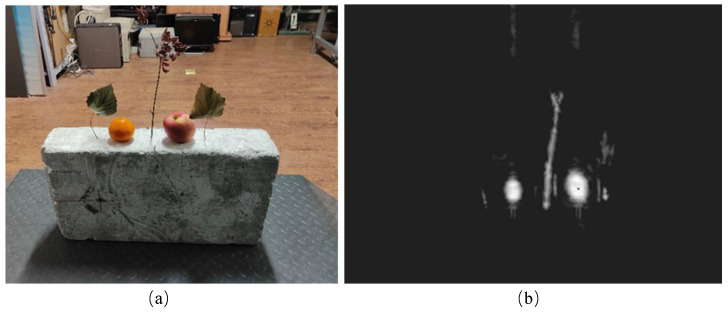
Comparison of RGB and Ka-band near-field MIMO-SAR imaging (**a**) RGB image, (**b**) Ka-band near-field MIMO-SAR image.

Accurate fruit detection represents a critical component of precision agriculture, facilitating reliable yield estimation, timely harvesting, and informed orchard management strategies [1,2]. While optical imagery has traditionally formed the backbone of these applications, its inherent dependence on illumination and weather conditions can limit data availability and quality [3]. In contrast, Synthetic Aperture Radar (SAR) imaging, with its capability to penetrate clouds and operate independently of daylight, provides consistent and robust data acquisition, making it particularly suited for continuous orchard monitoring [4]. A direct comparison of the same target captured by these two modalities (RGB vs. Ka-band near-field MIMO-SAR) is shown in Figure 1. However, applying state-of-the-art detection algorithms to SAR imagery for the purpose of apple detection remains a significant challenge due to the modality’s distinct imaging characteristics and the subtle nature of apple signatures within complex orchard canopies.

Several fundamental factors complicate apple detection in SAR data. First, unlike optical images that offer rich color, texture, and intensity gradients, SAR data represent scenes primarily through microwave backscatter intensities [5,6]. This results in low-contrast, texture-deficient imagery, where apples often appear as weak scatterers easily confounded by speckle noise, foliage clutter, and complex branching structures. Second, apples may vary substantially in size and distribution within the orchard, necessitating robust multi-scale feature extraction techniques that can discriminate between small, partially obscured targets and heterogeneous backgrounds [7,8]. Third, contextual cues—commonly exploited in optical imagery to provide additional semantic guidance—are not readily available in SAR data [9]. The identification of subtle relationships between targets and their surroundings becomes more demanding, requiring sophisticated approaches to incorporate both global and local contextual information [10].

Existing methods have sought to mitigate these challenges through advanced feature extraction and fusion strategies as well as attention mechanisms [11,12,13]. Multi-scale representations, such as those enabled by Spatial Pyramid Pooling (SPP), have proven effective in optical domains [14,15]. However, fixed-scale SPP configurations often fail to accommodate the dynamic variability present in SAR orchard scenes, leading to suboptimal scale selections for apple detection. Similarly, single-pass feature fusion techniques may struggle with the inherently noisy and semantically ambiguous nature of SAR features, resulting in unstable or semantically inconsistent representations that degrade detection performance [8]. Furthermore, while attention-based methods enhance feature discriminability by highlighting relevant regions or channels, a lack of explicit modeling of global and local contextual relationships may prevent the detector from leveraging crucial relational cues necessary for distinguishing subtle targets from complex backgrounds [16].

To address these gaps, this work introduces a novel detection framework tailored to the unique demands of SAR-based apple detection. The main contributions are threefold:

1. Dynamic Spatial Pyramid Pooling (DSPP): A learnable, adaptive pooling mechanism that replaces fixed-scale pooling with a dynamic selection process, emphasizing the most informative scales and spatial regions. This approach overcomes the rigidity of traditional SPP methods, enabling more effective multi-scale feature representation in SAR orchard imagery.
2. Recursive Feature Fusion Network (RFN): An iterative, recurrent-based fusion strategy that refines multi-level features over multiple passes. By repeatedly integrating information from various network layers, RFN stabilizes feature representations, enhances semantic coherence, and mitigates the impact of noise and low-contrast targets.
3. Context-Aware Feature Enhancement (CAFE): An attention-driven module that explicitly incorporates global and local contextual cues into the detection pipeline. CAFE models long-range dependencies and relational patterns, allowing the framework to exploit subtle contextual information that improves discrimination between apples and surrounding clutter.

These components are jointly optimized in an end-to-end framework, resulting in a detector that is robust, adaptive, and contextually aware. Experimental results demonstrate that the integrated DSPP-RFN-CAFE approach significantly improves detection accuracy and robustness compared to baseline methods, paving the way for more reliable SAR-based orchard monitoring and advancing the state of the art in remote sensing applications for precision agriculture [5].

## 2. Literature Review

### 2.1. SAR in Agricultural Remote Sensing and Near-Field Millimeter-Wave SAR

Synthetic Aperture Radar (SAR) has become an essential tool in agricultural remote sensing due to its capability for all-weather, day-and-night imaging [1]. Compared with optical methods, SAR can acquire consistent data under varying conditions, which is critical for reliable crop monitoring and yield forecasting [2]. In orchard environments, near-field millimeter-wave SAR has drawn attention because it achieves higher resolution at shorter ranges, enabling precise detection of fruits within complex canopies [3]. Recent technological advances in Multiple-Input Multiple-Output (MIMO) SAR have further enhanced near-field imaging performance by leveraging multiple transmit–receive channels to emulate a larger synthetic aperture [4,5]. This configuration not only provides high spatial resolution but also broad coverage suitable for fine-grained orchard monitoring tasks such as apple detection [6]. Despite its advantages, near-field millimeter-wave MIMO-SAR still faces challenges like increased clutter, speckle noise, and weak scattering signatures of fruits. Therefore, novel strategies for feature extraction and fusion are needed to harness the full potential of this technology in orchard scenarios.

### 2.2. Apple Detection

Apple detection has been widely studied in precision agriculture, primarily using optical imagery and conventional deep learning frameworks such as Faster R-CNN, YOLO, and RetinaNet [7,8,9]. While these methods perform well under favorable illumination and low occlusion, their reliance on visible-spectrum data often limits robustness to adverse conditions or dense foliage [10].

In contrast, SAR imagery is invariant to lighting and weather, making it a valuable complementary data source for fruit detection [11]. However, apples exhibit relatively weak backscattering in SAR images, and orchard scenes present complicated backgrounds with overlapping structures. Prior work has shown that multi-scale adaptation, feature fusion, and context modeling are crucial for accurate detection in such challenging conditions [12]. This study builds on these insights by integrating advanced modules—Dynamic Spatial Pyramid Pooling (DSPP), Recursive Feature Fusion Network (RFN), and Context-Aware Feature Enhancement (CAFE)—specifically tailored to address the unique characteristics of near-field millimeter-wave MIMO-SAR data.

### 2.3. Dynamic Spatial Pyramid Approaches

Multi-scale feature extraction is foundational in tasks where object sizes and scene complexity vary significantly. Classical Spatial Pyramid Pooling (SPP) introduced fixed-scale pooling partitions, enabling CNNs to handle variable input sizes [13]. However, static SPP methods often struggle with the highly diverse target distributions found in remote sensing and SAR data [14].

Dynamic Spatial Pyramid Pooling (DSPP) enhances adaptability by learning to emphasize or de-emphasize certain scales. Techniques incorporating attention mechanisms or learnable parameters can automatically adjust the number of pyramid levels, partition sizes, or scale ratios [15]. This level of flexibility becomes particularly valuable for near-field millimeter-wave SAR, where targets like apples may occupy varying proportions of the image and display weak scattering signatures.

### 2.4. Recursive Feature Fusion Approaches

Multi-level feature fusion has long been recognized as key to improving detection and segmentation accuracy [16]. Feature Pyramid Networks (FPN) and similar frameworks typically fuse features in a single pass, propagating semantic information downward to handle objects at different scales. Recursive Feature Fusion (RFF) extends this idea by iteratively refining multi-scale features over multiple interactions, analogous to how recurrent neural networks process temporal sequences [17]. Through repeated blending, the network can progressively suppress noise, clarify boundaries, and enhance feature stability. For near-field millimeter-wave SAR imaging, such iterative refinement helps isolate apple signatures from clutter and boosts overall detection robustness.

### 2.5. Context-Aware Feature Enhancement

Contextual cues often prove decisive in distinguishing targets from complex backgrounds. Traditional local descriptors can be insufficient when objects are partially occluded or visually similar to their surroundings [18]. Context-Aware Feature Enhancement (CAFE) techniques integrate information beyond the immediate neighborhood, capturing global relationships or long-range dependencies [19]. These methods frequently employ attention-based mechanisms or graph-based models to identify salient regions and unify scattered cues across the image [20]. In the context of near-field millimeter-wave SAR apple detection, embedding such context-aware modules can significantly mitigate weak scattering effects, reduce ambiguities, and reinforce target clues. By combining DSPP, RFN, and CAFE, our proposed framework aims to provide a more comprehensive and robust solution to the challenges of SAR-based fruit detection.

Context-Aware Feature Enhancement methods center on modeling relationships among pixels or feature points to embed local representations within a broader contextual framework. Key approaches include the following:

1. Attention-Based Context Modeling: Self-attention and non-local operations have emerged as powerful tools for capturing contextual cues [21]. By computing pairwise similarities among all spatial positions, attention mechanisms associate distant regions, identifying crucial contextual information spanning the entire image. Non-local Networks [22] and Criss-Cross Attention [19] exemplify methods that significantly improve contextual understanding in semantic segmentation and object detection tasks.
2. Graph-Based Context Integration: Another route involves representing features as graph nodes and employing Graph Convolutional Networks (GCNs) or Graph Attention Networks (GATs) to model relationships among nodes [23]. This approach aggregates scattered and weak features across the image, forming semantically meaningful structures by leveraging global contextual information.
3. Multi-Scale Context Fusion: Context does not only arise globally; it can also originate from neighboring local regions or mid-range structures. Techniques leveraging multi-scale context pooling or pyramid context modules, such as PSPNet [24] and the DeepLab series [25], integrate information across multiple spatial scales, reinforcing context representation from global to local levels.

In the SAR-based apple detection scenario, context-aware feature enhancement is especially pertinent. The inherently weak scattering signatures of apples in SAR imagery make it challenging to distinguish them from cluttered backgrounds through local features alone. By introducing a Context-Aware Feature Enhancement (CAFE) module, the network can gather discriminative contextual cues from the global image extent and reinforce statistical correlations between targets and their surroundings. This contextual integration is critical for suppressing noise, mitigating occlusions, and highlighting apples amid complex scattering environments. The CAFE module proposed in this study implements these principles, combining attention mechanisms and context modeling to achieve more robust apple detection in SAR imagery.

## 3. Data Collection and Imaging Methods

**Figure 2:**
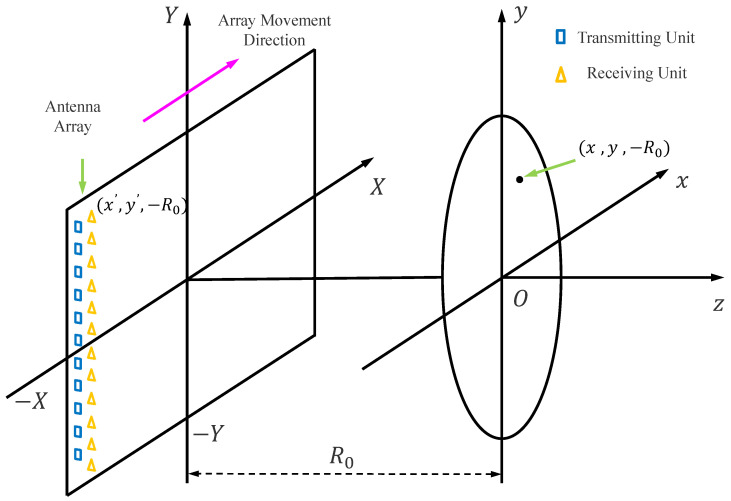
Schematic Diagram of the Ka-Band Near-Field MIMO-SAR Imaging System.

We employ a linear array mechanical scanning planar aperture near-field wideband three-dimensional imaging approach. As shown in Figure 2, taking a one-dimensional linear array scanning along the x-axis as an example, the antenna array is aligned parallel to the y-axis and includes both transmit and receive units operating in a monostatic mode. At each sampling position, signals are transmitted and received in sequence. A mechanical scanning device drives the antenna array to move steadily along the x-axis, thus forming a two-dimensional planar aperture. The center of the region to be imaged is set as the coordinate origin, and the z-axis is perpendicular to the planar aperture, representing the range direction.

Because the antenna array performs high-speed electronic scanning and the target is very close to the array, the horizontal movement during the vertical electronic scanning can be neglected. For each column of sampled data, it is assumed that the horizontal sampling positions remain constant. Under ideal conditions, we assume that the transmit and receive unit positions coincide [26,27,28]. Therefore, the ideal sampling positions corresponding to the two-dimensional aperture are $\left(x^{\prime },y^{\prime },-R_{0}\right)$. Let the coordinates of a target scattering point be $\left(x,y,z_{2}\right)$ and the distance from the scattering point to the antenna element is given by:

(1) $$R=\sqrt{{\left(x^{\prime }-x\right)}^{2}+{\left(y^{\prime }-y\right)}^{2}+{\left(R_{0}+z\right)}^{2}}$$

where σ(x,y,z) denotes the scattering coefficient of each target scattering point. According to the wavenumber domain imaging algorithm, for a planar aperture, the baseband echo signal can be expressed as:

(2) $$s\left(x^{\prime },y^{\prime },k\right)=\;\int \int \int \;\sigma \left(x,y,z\right)\exp\left[-j2k\sqrt{{\left(x^{\prime }-x\right)}^{2}+{\left(y^{\prime }-y\right)}^{2}+{\left(R_{0}+z\right)}^{2}}\right]dxdydz$$

where $k=\frac{2\pi f}{c}$, *f* represents the operating frequency (or frequency band if multiple frequencies are used), and c denotes the speed of electromagnetic wave propagation in free space. The product 2πf/c gives the fundamental wavenumber for the imaging process.

Based on the wavenumber domain imaging algorithm [3], the target scattering coefficient distribution can be obtained by mapping $s(x^{\prime },y^{\prime },k)$ into the $\left(k_{x},k_{y},k\right)$ domain via two-dimensional Fourier transforms in $x^{\prime }$ and $y^{\prime }$. Once the data are expressed as $s(k_{x},k_{y},k)$, it becomes possible to perform a three-dimensional reconstruction via Stolt interpolation. Specifically,

(3) $$\sigma \left(x,y,z\right)=\;\int \int \int \;s\left(k_{x},k_{y},k\right)\exp\left\lceil j\left(k_{x}x+k_{y}y+\sqrt{4k^{2}-k_{x}^{2}-k_{y}^{2}}\left(R_{0}+z\right)\right)\right]dk_{x}dk_{y}dk^{2}$$

where $R_{0}$ is the vertical distance from the array to the coordinate origin. In practical imaging applications, $R_{0}$ can be chosen according to the imaging range and other factors.

According to the dispersion relation, the signal wavenumber *k* and the spatial wavenumber components $k_{x}$, $k_{y}$, and $k_{z}$ satisfy the following:

(4) $$k_{x}^{2}+k_{y}^{2}+k_{z}^{2}=4k^{2},k_{z}=\sqrt{4k^{2}-k_{x}^{2}-k_{y}^{2}}$$

To perform a three-dimensional inverse Fourier transform, $s(k_{x},k_{y},k)$ must be interpolated onto a uniformly distributed grid in the $\left(k_{x},k_{y},k_{z}\right)$ coordinate system, resulting in $s(k_{x},k_{y},k_{z})$. This step is known as Stolt interpolation [29]. After interpolation, we obtain $s(k_{x},k_{y},k_{z})$, which is uniformly distributed in the frequency domain. A three-dimensional inverse Fourier transform then yields the target scattering coefficient distribution σ(x,y,z).

The steps of the wavenumber domain algorithm are described below.

First, a two-dimensional Fourier transform is applied along the horizontal and vertical dimensions of the echo signal $s\left(x^{\prime },y^{\prime },k\right)$ to obtain the frequency domain signal $s\left(k_{x},k_{y},k\right)$. This frequency-domain signal is then multiplied by the reference function exp $\left[j\sqrt{4k^{2}-k_{x}^{2}-k_{y}^{2}}R_{0}\right]$ to compensate for the propagation phase. Next, Stolt interpolation is performed to map $s\left(k_{x},k_{y},k\right)$ to $s\left(k_{x},k_{y},k_{z}\right)$. Finally, a three-dimensional inverse Fourier transform of $s\left(k_{x},k_{y},k_{z}\right)$ yields the target scattering coefficient distribution σ(x,y,z). To provide a clearer mathematical expression, σ(x,y,z) can be written as:

(5) $$\sigma \left(x,y,z\right)={\mathrm{FT}}_{3D}^{-1}\left[{\mathrm{IN}}_{1D}\left({\mathrm{FT}}_{2D}\left[s\left(x^{\prime },y^{\prime },k\right)\right]\exp\left(j\sqrt{4k^{2}-k_{x}^{2}-k_{y}^{2}}R_{0}\right)\right)\right]$$

where ${\mathrm{FT}}_{2D}$ denotes the two-dimensional Fourier transform in the horizontal and vertical directions ($x^{\prime }$ and $y^{\prime }$), ${\mathrm{IN}}_{1D}$ is the one-dimensional Stolt interpolation mapping {k} to $\left\{k_{z}\right\}$, and ${\mathrm{FT}}_{3D}^{-1}$ represents the three-dimensional inverse Fourier transform. Different parts of the target exhibit distinct reflection coefficients; once the three-dimensional scattering distribution σ(x,y,z) is obtained, target imaging can be readily performed.

### 3.1. Prototype Composition

**Figure 3:**
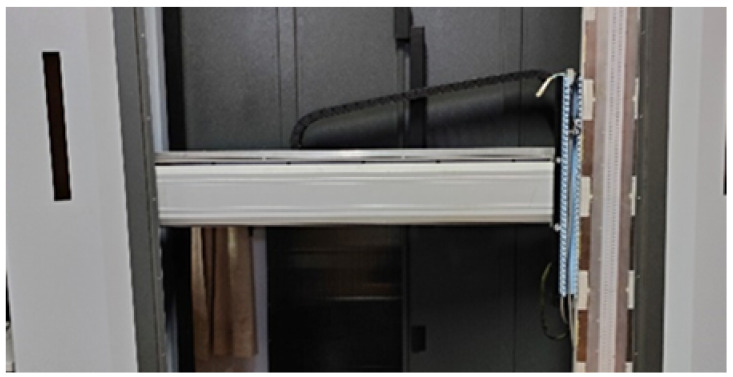
Mechanical Scanning Device for the Ka-Band Near-Field MIMO-SAR Imaging System.

The experimental system mainly consists of four parts: the millimeter-wave array, the mechanical scanning device, the control system, and the data acquisition and processing system. The radar transmits Ka-band signals (with wavelengths ranging from 7.5 mm to 11.25 mm). The millimeter-wave array module integrates an antenna array, switching arrays, RF channels, and frequency synthesis modules. The mechanical scanning device moves the millimeter-wave array horizontally and provides real-time feedback on the array’s horizontal position. The control system coordinates all components, controlling the mechanical scanning for horizontal movement, the millimeter-wave array module for signal transmission and reception, and the data acquisition and processing system for data collection and final image output. An image of the mechanical scanning device is shown in Figure 3.

### 3.2. Experimental System Configuration and Parameter Settings

#### 3.2.1. Prototype Parameters

Table 1 lists the parameters of the Ka-band near-field wideband three-dimensional imaging prototype. A linear frequency-modulated (LFM) signal is transmitted, with frequencies ranging from 32 GHz to 37 GHz. In practical applications, when the subject under inspection is close to the array and the array aperture exceeds the antenna element beam coverage, the azimuth resolution of the entire array’s full-resolution area is determined by the antenna element’s beamwidth. Additionally, because the target occupies a relatively small range, the range window can be appropriately narrowed. Under acceptable signal-to-noise ratios, the echo data can be suitably downsampled [4].

#### 3.2.2. Experimental Dataset Acquisition

We collaborated with the Beijing Institute of Technology and the First Research Institute of the Ministry of Public Security to use a MIMO-SAR-based millimeter-wave imaging device for experimental exploration. The experimental results indicate that the device can complete fruit tree scanning and imaging within 10 s. Using the aforementioned experimental setup, we conducted data acquisition experiments.

**Figure 4:**
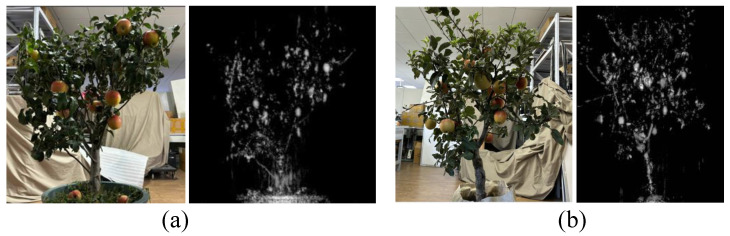
Two apple tree imaging samples from our experiments, shown in subfigures (**a**,**b**). In each subfigure, the left panel is the RGB image, and the right panel is the Ka-band SAR image.

Two real fruit-bearing trees were placed at a distance of about 30 cm directly in front of the experimental platform. The scanning mode was enabled, and the test interface is shown in Figure 4a. By continuously rotating the fruit trees and scanning from different angles, SAR images were obtained and stored once the test was completed. A smartphone mounted behind the device captured corresponding optical images after the SAR scanning finished, producing paired samples of optical and SAR images. In total, we collected 150 pairs of samples, covering about 15 fruit trees, yielding approximately 2000 SAR fruit samples. Figure 4b shows an example.

Table 1 outlines the specifications of a prototype system for near-field broadband 3D imaging in the Ka band.

## 4. Method

This section describes the proposed methodology for detecting apples in Synthetic Aperture Radar (SAR) imagery, a task complicated by low signal-to-noise ratios, lack of intuitive texture or color cues, and the presence of speckle noise and clutter. To address these issues, the method integrates three key components: (1) Dynamic Spatial Pyramid Pooling (DSPP) for adaptive multi-scale feature extraction, (2) a Recursive Feature Fusion Network (RFN) for iterative multi-level feature refinement, and (3) a Context-Aware Feature Enhancement (CAFE) module for leveraging global and local contextual relationships. Together, these components form a detection framework suitable for handling the subtle and often low-contrast appearance of apples in SAR images, ultimately improving robustness and accuracy in challenging orchard environments.

### 4.1. Problem Setup

Let $I\in R^{H\times W}$ denote an input SAR image. The objective is to predict the positions and extents of apples within this image. An initial feature extraction step is performed using a deep convolutional backbone network (e.g., a ResNet variant), yielding a feature map $F\in R^{C\times H^{\prime }\times W^{\prime }}$. This feature map serves as the input for the subsequent modules described below. The overall pipeline is end-to-end trainable, enabling joint optimization of all parameters with respect to a defined detection objective (e.g., a combination of classification and regression losses).

### 4.2. Dynamic Spatial Pyramid Pooling (DSPP)

**Figure 5:**
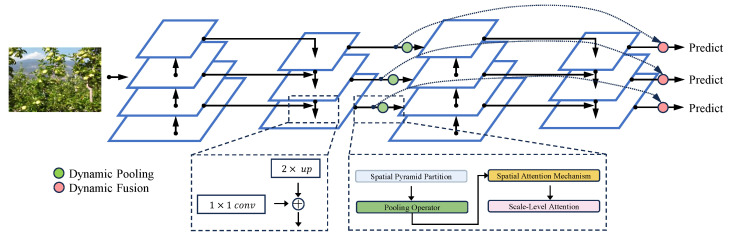
Block Diagram of the Dynamic Spatial Pyramid Pooling (DSPP) Module.

In standard computer vision tasks, Spatial Pyramid Pooling (SPP) provides multi-scale representations by pooling features over increasingly fine subregions of the feature map. However, traditional SPP relies on predetermined scaling levels, which may not be optimal for SAR images of apple orchards characterized by widely varying target scales, cluttered backgrounds, and strong speckle noise. As illustrated in Figure 5, the proposed DSPP module introduces trainable attentional mechanisms to adaptively select the most informative scales and spatial regions.

The DSPP module introduces adaptability into the spatial pyramid. Consider *L* pyramid levels, each partitioning the feature map F into a grid of $R_{l}\times R_{l}$ cells at level l. For each cell, a pooling operator (e.g., max or average pooling) aggregates the features:

(6) $$x_{r,c}^{\left(l\right)}=\mathrm{Pool}\left(F_{r,c}^{\left(l\right)}\right),$$

where $F_{r,c}^{\left(l\right)}$ represents the subregion of F corresponding to the (r,c)-th cell at the *l*-th pyramid level. To introduce adaptability, trainable weights $\alpha_{l}$ are used to produce normalized attention coefficients $W_{l}$ through a softmax function:

(7) $$W_{l}=\frac{\exp\left(\alpha_{l}\right)}{\sum_{k=1}^{L}\exp\left(\alpha_{k}\right)}$$

Additionally, within each pyramid level, a spatial attention mechanism assigns attention scores $a_{r,c}^{\left(l\right)}$ to emphasize informative regions:

(8) $$a_{r,c}^{\left(l\right)}=\frac{\exp\left(\emptyset \left(x_{r,c}^{\left(l\right)}\right)\right)}{\sum_{r^{\prime },c^{\prime }}\exp\left(\emptyset \left(x_{r^{\prime },c^{\prime }}^{\left(l\right)}\right)\right)}$$

where ∅(·) is a learnable mapping. The DSPP output is formed as a weighted aggregation of the level-wise attention-pooled features:

(9) $$X_{\mathrm{DSPP}}=\sum_{l=1}^{L}W_{l}\left(\sum_{r=1}^{R_{l}}\sum_{c=1}^{R_{l}}a_{r,c}^{\left(l\right)}x_{r,c}^{\left(l\right)}\right)$$

This dynamic formulation enables the model to emphasize the most appropriate scales and spatial regions for apple detection in SAR images.

### 4.3. Recursive Feature Fusion Network (RFN)

**Figure 6:**
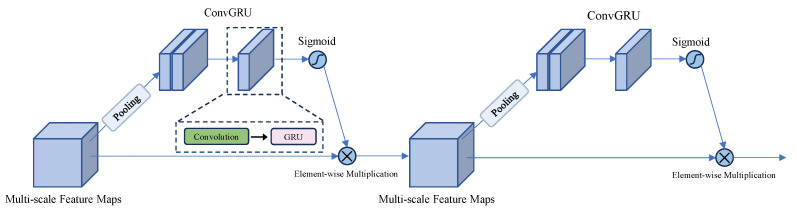
Block Diagram of the Near-Field Recursive Feature Fusion Network (RFN).

While DSPP provides adaptive multi-scale representations, single-pass feature fusion may be insufficient for SAR data, which often exhibit weak contrasts, non-distinctive textures, and significant noise. Therefore, we introduce a Recursive Feature Fusion Network (RFN) to iteratively refine multi-level features, leading to more stable and coherent representations. As illustrated in Figure 6, the RFN aggregates feature maps from multiple network stages in a recurrent manner.

Let ${\left\{F_{m}\right\}}_{m=1}^{M}$ denote a set of feature maps extracted from *M* different network stages or scales. In practice, *M* corresponds to the number of multi-level feature maps that the backbone network outputs. In this study, ResNet-50 provides four principal feature scales (C2, C3, C4, and C5), and thus, M=4. This choice balances detection performance and computational overhead, though additional levels can be included when higher resolution or more nuanced feature representation is required. Initially, a fusion operator R(·) aggregates these features:

(10) $$F_{\mathrm{fusion}}^{\left(1\right)}=R\left({\left\{F_{m}\right\}}_{m=1}^{M}\right)$$

Rather than relying on a single-shot integration, the RFN applies multiple refinement iterations. At iteration *t*:

(11) $$F_{\mathrm{fusion}}^{\left(t\right)}=R\left(F_{\mathrm{fusion}}^{\left(t-1\right)},{\left\{F_{m}\right\}}_{m=1}^{M}\right)$$

Implementations often employ recurrent layers such as ConvGRU or LSTM-inspired blocks adapted for feature maps. After *T* iterations, the final recursive fusion output is as follows:

(12) $$F_{\mathrm{RFN}}=F_{\mathrm{fusion}}^{\left(T\right)}$$

This iterative process allows low-level and high-level features to be repeatedly integrated, mitigating the effects of noise and strengthening the semantic consistency of the resulting representation, which is critical for accurately identifying small or partially obscured apples in SAR images.

### 4.4. Context-Aware Feature Enhancement (CAFE)

**Figure 7:**
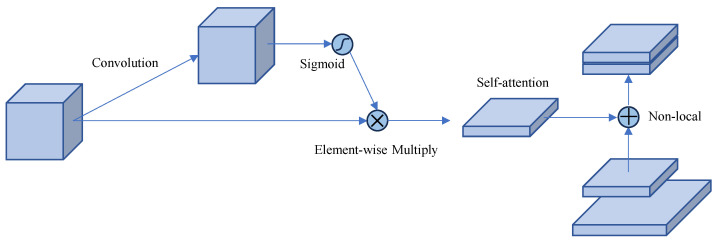
Block Diagram of the Context-Aware Feature Enhancement (CAFE) Module.

Even after multi-scale adaptation and iterative fusion, detection in SAR imagery may still benefit from incorporating contextual information. Apples often appear as small, isolated reflectors embedded in complex background structures. As illustrated in Figure 7, the CAFE module introduces global and local contextual modeling to enhance feature discrimination, particularly in noisy and cluttered orchard environments.

This work adopts a self-attention mechanism to capture long-range dependencies. Starting from $F_{\mathrm{RFN}}\in R^{c\times H^{\prime }\times W^{\prime }}$, the spatial dimensions are flattened to $HW^{\prime }$. Three linear transformations are applied:

(13) $$\theta \left(F_{\mathrm{RFN}}\right)=W_{\theta }F_{\mathrm{RFN}},\phi \left(F_{\mathrm{RFN}}\right)=W_{\phi }F_{\mathrm{RFN}},g\left(F_{\mathrm{RFN}}\right)=W_{q}F_{\mathrm{RFN}}$$

where $F_{j}$ denotes the *j*-th position (or spatial location) in $F_{\mathrm{RFN}}$. The term $g\left(F_{j}\right)$ is a learnable mapping that enriches the feature representation for attention-based context aggregation. A similarity function, as follows:

(14) $$f\left(F_{i},F_{j}\right)=\frac{\exp\left(\theta {\left(F_{i}\right)}^{⊤}\phi \left(F_{j}\right)\right)}{\sum_{j}\exp\left(\theta {\left(F_{i}\right)}^{⊤}\phi \left(F_{j}\right)\right)},$$

measures the similarity between locations *i* and *j*. Subsequently, contextual aggregation is performed:

(15) $$Y_{i}=\sum_{j}f\left(F_{i},F_{j}\right)g\left(F_{j}\right)$$

Reshaping *Y* back to the original dimensions and adding it to $F_{\mathrm{RFN}}$ yields the enhanced features:

(16) $$F_{\mathrm{CAFE}}=F_{\mathrm{RFN}}+Y$$

This operation incorporates context-driven cues, helping to highlight subtle apple signatures against complex backgrounds.

### 4.5. Detection Head and Training Procedure

The final enhanced representation $F_{\mathrm{CAFE}}$ is forwarded to a detection head (e.g., anchor-based or anchor-free) to predict bounding boxes and classification scores. Standard loss functions such as a combination of focal loss (for classification) and Smooth L1 loss (for bounding box regression) are employed.

Training proceeds end-to-end. The backbone, DSPP, RFN, CAFE, and detection head parameters are jointly optimized via stochastic gradient-based methods (e.g., SGD or Adam). Appropriate data augmentation and regularization strategies are applied to improve generalization. During inference, the trained model processes a given SAR image through the entire pipeline and applies non-maximum suppression to produce the final apple detections.

## 5. Experiments and Results Analysis

This section presents a comprehensive evaluation of the proposed near-field millimeter-wave SAR apple detection framework, which integrates Dynamic Spatial Pyramid Pooling (DSPP), a Recursive Feature Fusion Network (RFN), and the Context-Aware Feature Enhancement (CAFE) module. We begin by introducing the dataset and evaluation metrics employed in our experiments. We then compare the proposed approach with several representative object detection models, followed by an ablation study that examines the individual contributions and underlying mechanisms of each module. Additionally, we provide qualitative analyses, assess computational efficiency, and discuss potential future research directions for leveraging SAR data in precision agriculture fruit detection and yield estimation.

### 5.1. Dataset and Evaluation Metrics

The dataset used in this study was acquired using the near-field millimeter-wave MIMO-SAR imaging system described in Section 3. It contains approximately 150 paired SAR-optical image samples and around 2000 accurately annotated apple targets. During data collection, we varied the viewing angles by rotating the fruit-bearing trees and performing multi-angle scanning to ensure diversity and complexity. As a result, the dataset encompasses significant variability in apple size, shape, spatial distribution, and background scattering characteristics, effectively simulating the diverse conditions encountered in real-world orchard environments.

To facilitate objective performance evaluation, the dataset was split into training and test sets at a ratio of roughly 7:3. The training set, with its rich variety of samples, supports robust parameter learning and improved generalization, while the test set serves as an unbiased benchmark of final detection performance. During annotation, corresponding optical images were leveraged to accurately determine apple positions and sizes in the SAR imagery, ensuring annotation precision and consistency.

In terms of evaluation metrics, we adopt the commonly used Average Precision (AP) measure in object detection, computed at an IoU (Intersection over Union) threshold of 0.5. AP effectively quantifies how well predicted bounding boxes align with ground truth targets. In addition, we report Recall and F1-score to provide a more holistic assessment of detection performance under different precision–recall trade-offs.

### 5.2. Baseline Models and Experimental Setup

To validate the effectiveness of our proposed framework, we compared it against several classical and widely used object detection models that serve as baseline references. The first model, Faster R-CNN [1], is a classic two-stage detector renowned for its success in optical imagery. Applying this model directly to SAR data establishes a lower bound on performance within this challenging domain. The second set of models, YOLOv3 and YOLOv5 [2,3], are representative single-stage, real-time detectors known for their speed and adaptability across multiple scales. Evaluating these models on SAR data, which often exhibits weak textures, provides insights into the stability and robustness of single-shot methods under complex conditions.

The third model, RetinaNet with a Feature Pyramid Network (FPN) [4], is a single-stage detector that integrates FPN for multi-scale feature fusion and employs Focal Loss to address class imbalance during training. Its proficiency in detecting small objects makes it a relevant benchmark for assessing our multi-scale approach. Additionally, we incorporated Faster R-CNN with a Static Spatial Pyramid Pooling (SPP) module, which adds a traditional fixed-scale SPP to Faster R-CNN. This setup serves as a baseline for evaluating the effectiveness of a static multi-scale strategy on SAR data, providing a point of comparison for our dynamic DSPP approach.

All models were implemented using the PyTorch (version 2.0.0, developed by Meta AI, Menlo Park, CA, USA) framework and trained and tested on a workstation equipped with an NVIDIA GPU. To enhance model generalization, we employed various data augmentation techniques, including horizontal flipping, translation, and noise perturbation. For optimization, we utilized either SGD or Adam optimizers, initially increasing the learning rate gradually through a warm-up strategy. In the later stages of training, the learning rates were adjusted using step decay or cosine annealing to ensure stable convergence.

### 5.3. Quantitative Experiments

In order to evaluate the performance of different detection models on near-field MIMO-SAR apple detection, we measure their AP, Recall, F1-score, and average inference time under the same hardware conditions (e.g., an NVIDIA Tesla V100 with single-image inference). Table 2 provides a comprehensive summary of these metrics for both classical optical-based detectors (Faster R-CNN, YOLOv3, YOLOv5, and RetinaNet) and two SAR-specific methods (LSDN-RC and YOLO-RC), along with our proposed DSPP + RFN + CAFE approach. The results reveal that standard models such as Faster R-CNN and YOLO variants typically yield AP values around 60–65%, indicating that weak-texture apple targets and cluttered backgrounds pose significant challenges for conventional detectors that depend heavily on visual priors. Although RetinaNet with FPN and Faster R-CNN supplemented by a static SPP module achieve moderate performance gains, their AP improvements remain relatively limited.

Meanwhile, LSDN-RC and YOLO-RC—both tailored to the range-compressed SAR domain—demonstrate improved performance over purely optical-based methods, with AP in the 69–72% range. However, they still fall short of effectively mitigating the weak-reflector problem inherent in near-field orchard imaging. In contrast, our proposed DSPP + RFN + CAFE framework achieves a remarkable AP of 81.6%, surpassing all competing models by at least 10 percentage points. Such a significant improvement underscores the effectiveness of dynamic multi-scale pooling (DSPP), iterative feature refinement (RFN), and context-aware enhancement (CAFE) in addressing the low-contrast, noise-prone conditions characteristic of SAR-based apple detection. This substantial performance boost paves the way for more reliable fruit monitoring in precision agriculture.

### 5.4. Ablation Study

To investigate the contribution of each module, we conducted an ablation study by progressively adding or removing DSPP, RFN, and CAFE, with the results presented in Table 3. Incorporating DSPP alone increased the Average Precision (AP) from 64.1% to 69.0%, affirming that a dynamic multi-scale strategy is crucial for handling the diverse scale variations in SAR orchard scenes. Introducing RFN on top of DSPP further raised the AP to 75.2%, demonstrating that recursive feature fusion effectively refines feature representations and enhances stability and semantic consistency amid weak textures. Similarly, combining CAFE with DSPP yielded an AP of 73.5%, illustrating that context-aware enhancement, through modeling global and local dependencies, provides essential semantic cues for robust detection. When DSPP, RFN, and CAFE were employed jointly, the AP achieved an impressive 81.6%, representing a remarkable 17.5-point improvement over the baseline. This significant enhancement confirms that the three modules complement each other, collectively boosting feature representation and contextual understanding to achieve breakthrough performance in SAR-based apple detection. Table 3 illustrates the improvements in average precision (AP) percentage with various configurations of the detection model, showcasing the effectiveness of each component in enhancing the overall detection accuracy.

### 5.5. Qualitative Analysis and Visualization

**Figure 8:**
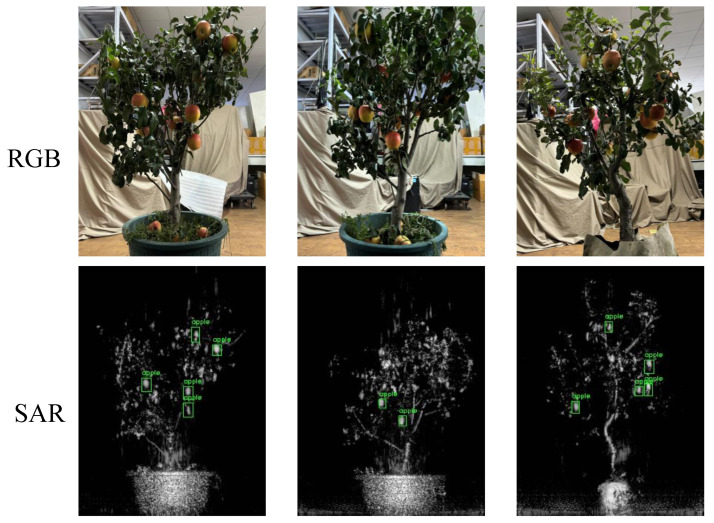
Qualitative results of object detection.

We conducted qualitative comparisons on selected test samples as shown in Figure 8. Unlike traditional methods, such as Faster R-CNN, which often miss or misclassify weak-scattering targets, our framework produces more precise bounding boxes. Even in challenging conditions involving occlusion by leaves, complex backgrounds, or subtle scattering signatures, our method reliably locates apples. This performance gain is driven by three key factors: DSPP, which dynamically emphasizes scales relevant to various apple sizes; RFN, which iteratively refines features to suppress noise and ambiguity; and CAFE, which integrates context to leverage environmental cues within complex scenes. Together, these components enhance the framework’s ability to accurately detect apples in diverse and intricate SAR-based orchard environments.

### 5.6. Runtime Efficiency and Practical Implications

In practical agricultural applications, detection speed and energy efficiency are also important considerations. Although our method introduces a higher computational load compared to single-stage detectors, inference on a GPU-accelerated workstation still occurs within hundreds of milliseconds per SAR image. Future work could involve model pruning, distillation, quantization, or parallelization to meet large-scale and near-real-time monitoring demands.

This study offers a new technical pathway for fruit detection and yield estimation using near-field millimeter-wave SAR data. With ongoing advancements in imaging hardware, computational resources, and the potential fusion of optical or multi-spectral data, there is a promising future for comprehensive, automated fruit detection and decision support systems in precision agriculture.

### 5.7. Summary

In this section, we validated the effectiveness of our proposed approach on a dedicated near-field millimeter-wave SAR apple dataset. Compared to various classical and state-of-the-art detectors, our method achieves a pronounced lead in AP. Ablation studies confirmed the synergistic effects of DSPP, RFN, and CAFE in enabling dynamic multi-scale adaptation, iterative feature refinement, and context-aware modeling. Qualitative analyses further substantiated the framework’s ability to accurately identify apple targets amidst challenging scattering backgrounds. Overall, this work provides a valuable reference for high-precision fruit detection under stringent SAR imaging conditions and establishes a theoretical and experimental foundation for adopting such technologies in precision agriculture practices.

## 6. Conclusions

This study addresses the challenges of apple detection in SAR imagery, characterized by weak scattering, low contrast, and complex backgrounds, by proposing an integrated detection framework that incorporates Dynamic Spatial Pyramid Pooling (DSPP), a Recursive Feature Fusion Network (RFN), and a Context-Aware Feature Enhancement (CAFE) module. Through in-depth analysis and empirical validation, we have drawn several key conclusions and contributions:

- Effectiveness of Dynamic Multi-Scale Representations: The DSPP module overcomes the limitations of traditional fixed-scale spatial pyramid pooling. By introducing adaptive and learnable mechanisms, it highlights the most informative scales and regions of the image. This adaptability significantly improves the model’s robustness to varying apple sizes and distributions within the complex SAR orchard environment.
- Refinement Through Recursive Feature Fusion: The RFN module employs iterative feature integration, progressively refining features to mitigate noise and semantic uncertainty. Compared to a single-pass fusion, this recursive approach stabilizes feature representations and enhances semantic clarity, resulting in more reliable detection performance under low signal-to-noise conditions.
- Enhancing Discriminative Power With Context-Aware Modeling: The CAFE module explicitly integrates global and local contextual information into the detection pipeline, compensating for the lack of intuitive texture and color cues in SAR images. By modeling both long-range dependencies and localized relationships, the module leverages environmental cues to improve the distinction between apples and cluttered backgrounds.

Extensive experiments on a dedicated near-field millimeter-wave SAR apple dataset demonstrate that our proposed framework significantly outperforms baseline methods, achieving substantial improvements in AP, Recall, and F1-score. Ablation studies confirm the synergistic benefits of DSPP, RFN, and CAFE in terms of multi-scale adaptability, iterative feature refinement, and context-aware modeling. Qualitative analyses further validate the capability of our approach to consistently detect apples, even under challenging conditions such as foliage occlusion and weak scattering signatures. In summary, this research provides a promising technical pathway for realizing high-precision fruit detection and yield estimation using SAR data, paving the way for future integration with advanced imaging modalities and data fusion strategies. The work lays a solid theoretical and experimental foundation for the adoption of SAR-based detection solutions in precision agriculture, ultimately contributing to more efficient orchard management and improved agricultural productivity.

## Figures and Tables

**Table 1:** Parameters of the Ka-band near-field wideband 3D imaging prototype.

Parameter	Value
Signal Frequency (GHz)	32∼37
Scanning and Imaging Method	Single-station linear array mechanical scanning synthetic aperture
Aperture Size (m)	1×1.92 (Horizontal × Vertical)
Antenna Unit Beam Width E/H plane (°)	55
Polarization Mode	Vertical linear polarization
Horizontal Sampling Interval (mm)	4
Vertical Sampling Interval (mm)	5

**Table 2:** Quantitative comparison of detection models. **Bold** values indicate the best performance.

Model	AP (%)	Recall (%)	F1-Score (%)	Inference Time (ms)
Faster R-CNN 1	62.3	67.1	64.6	34
YOLOv3 2	61.5	65.2	63.3	28
YOLOv5 3	63.0	68.0	65.4	20
RetinaNet(FPN) 4	65.4	69.5	67.4	25
Faster R-CNN + Static SPP	67.2	70.3	68.7	36
LSDN-RC [30]	69.1	72.3	70.7	23
YOLO-RC [31]	71.6	74.4	73.0	26
Ours (DSPP + RFN + CAFE)	**81.6**	**83.2**	**82.4**	**44**

**Table 3:** Ablation study results.

Model Configuration	AP (%)	Improvement Over Base Model (%)
Base Model (DSPP/RFN/CAFE)	64.1	-
Base Model + DSPP	69.0	+4.9
Base Model + DSPP + RFN	75.2	+11.1
Base Model + DSPP + CAFE	73.5	+9.4
Base Model + DSPP + RFN + CAFE (Complete Model)	81.6	+17.5

## Data Availability

The data presented in this study are available on request from the corresponding author.
